# GplusE: beyond genomic selection

**DOI:** 10.1002/fes3.52

**Published:** 2015-03-25

**Authors:** Ian Mackay, Eric Ober, John Hickey

**Affiliations:** ^1^ John Bingham LaboratoryNIABHuntingdon RoadCambridgeCB3 0LEUK; ^2^ The Roslin Institute and Royal (Dick) School of Veterinary StudiesUniversity of EdinburghEaster Bush Research CentreMidlothianEH25 9RGUK

**Keywords:** Genomic selection, high throughput phenotyping, phenomics, selection index, wheatwheat

## Abstract

GplusE is a strategy for genomic selection in which the accuracy of assessment in the reference population for a primary trait such as yield is increased by the incorporation of data from high‐ throughput field phenotyping platforms. This increase in precision comes from both exploiting genetic relationships between traits and reducing the effect of environmental influences upon them. We describe a collaborative project among researchers and breeders to develop a large reference population of elite UK wheat lines. This will be used to test the method, to study the design of the reference population, and to test genotyping strategies and imputation methods. Finally, it will provide data to pump‐prime the application of genomic selection to UK winter wheat breeding.

## Introduction

In this paper, we argue that the collection of high‐throughput phenotype data from field trials (HTP) (Montes et al. 2007; Araus and Cairns 2014) can be integrated directly into plant breeding programs in a manner analogous to the use of high‐density marker data in genomic selection (GS) (Meuwissen et al. 2001; Jannink et al. 2010). We focus on our own plans to integrate HTP with genomic prediction for UK winter wheat. To this end, we first give a brief overview of GS, then describe how HTP and GS can be brought together to improve response to selection. This combined approach we refer to as GplusE: in acknowledgment of the work of Johannsen (1857–1927) who first described phenotype as a function of genotype and environmental factors: an insight which still underpins plant breeding. We suggest that this approach can be extended to incorporate other sources of high throughput data into genomic prediction, for example, from metabolomics or gene expression experiments, and can also be used to improve the accuracy of field trials in general.

The rate of genetic improvement depends on four factors: (1) the accuracy of selection; (2) the time taken to generate new lines; (3) the number of selection candidates and proportion selected (selection intensity); (4) the genetic variability among the candidates for selection (Hallauer and Darrah 1985; Falconer and Mackay 1996). In wheat, most recent research effort has been placed on the fourth of these through projects that are curating and exploiting novel sources of genetic variation from landraces and wild species. For example, in the United Kingdom, £12 m is being invested by the BBSRC over 6 years from 2011 in a large collaborative project to introgress novel sources of germplasm into hexaploid wheat from crosses between adapted lines, 4× and 2× related species, and landraces (Galushko and Gray 2014; www.wheatisp.org). Similarly, the Mexican government invested $70 m into “Seeds of Discovery” at CIMMYT (Wenzl 2013; www.seedsofdiscovery.org), a 7‐year project to characterize wheat and maize germplasm. In recent years, however, the development of high throughput systems to record large quantities of genetic and phenotypic information cheaply presents other opportunities for increasing the rate of genetic improvement. In the case of genetics, high‐density genetic markers can be used directly to predict traits without recourse to initial QTL mapping experiments, leading to genomic selection (Meuwissen et al. 2001). We propose that in an analogous manner, measurements from high‐ throughput phenotyping systems can also be used in trait prediction without recourse to physiological or biochemical hypothesis testing or interpretation of those measurements (though these can help).

## Genomic Selection

In breeding programs, a major application for high‐density genetic markers in plant and animal breeding is to predict traits for individuals (Scutari etet al. 2013; Lin et al. 2014). These predicted traits can then be used in place of direct phenotyping to select among individuals. Since selection is no longer constrained by the time required to develop lines and to bulk up seed for phenotyping, rates of response to selection can be greatly increased. The optimum method of implementing genomic selection in any breeding program is an active area of research and varies with species and target traits (Jonas and de Koning 2013) but its use in commercial animal breeding is now well established (Hayes et al. 2013).

The principle of genomic selection is straight forward: a large population of individuals (the reference or training population) is phenotyped accurately and genotyped with a high‐density of genetic markers. Traits are regressed against markers to generate a prediction equation for the traits from the markers. Candidates for selection, which are not part of the reference population (RP), are genotyped and selected on the basis of their predicted trait values alone. Selected individuals can then be crossed and their progeny immediately genotyped in turn to enable selection to be performed amongst them. GS thus enables high intensities of selection (because large populations can be raised and genotyped at lower costs than when phenotyping is required) and a rapid cycle time (because candidates for selection do not need to be phenotyped). However, the devil is in the detail: in particular, in development of appropriate prediction algorithms, the number of markers required, and the composition and size of the RP. We comment briefly on each of these in turn.

### Prediction algorithms

Algorithm development for trait prediction is no longer viewed as critical because most methods give similar prediction accuracies in most situations (e.g., Daetwyler et al. 2013). The core statistical problem is that, with more markers than individuals, least squares regression methods do not work and statistical models that treat markers as random effects are needed (Whittaker et al. 2000; Meuwissen et al. 2001). Several of these methods (e.g., GBLUP, BayesA, BayesB, Bayes Lasso, Elastic Net) are implemented in freely available software (e.g., BGLR: Pérez and de los Campos 2014; AlphaBayes: Hickey and Tier 2009; GenSel: Fernando and Garrick 2009; ASReml: Gilmour et al. 2009) but perhaps the simplest method “GBLUP” is most widely used in practice because it is easy to implement, fast and gives results that are generally as good as those obtained with other methods (e.g., Daetwyler et al. 2013).

### Marker numbers

GS can require genotyping of thousands of selection candidates in each generation, with potentially several generations per year. Consequentially, even though cheap high‐throughput genotyping platforms are available for the major crops, cost can still be a constraint. There are two approaches to its control.

Firstly, GS can be limited to cases for which only low densities of markers are required. For example, cycles of genomic prediction and selection among individuals within a single cross require only a small reference population of lines from the same cross and only small numbers of markers. In this case, linkage disequilibrium (correlation between pairs of loci) extends over large genetic distances along the chromosomes and accurate predictions can be made from very small numbers of markers, potentially <100 (Hickey et al. 2014). However, making repeated cycles of selection within a cross is not the best breeding strategy; greater progress is made by crossing selected individuals from different crosses.

As relationships between the RP and the selection candidates decrease, marker density must also increase. There may be no recent pedigree relationship between candidates and the RP and in these cases high marker densities are required, of the order of tens of thousands (Hickey et al. 2014). A second approach in these circumstances is to control genotyping costs by marker imputation. Here, the RP is genotyped with a full set of markers but selection candidates are genotyped with a smaller selected subset. The pattern of linkage disequilibrium among markers in the RP is then used to predict genotypes for the missing markers for the candidates. This is standard practice in animal breeding where, for example, as many as 600,000 markers or whole genome sequence may be genotyped in the RP but as few as 384 markers on the candidates (e.g., Huang et al. 2012; Hickey et al. 2011; Habier et al. 2009; VanRaden et al. 2010). The cost reduction is therefore substantial. The process is less well developed in plant breeding; methods and software may need to be developed to account for complexity arising from large repetitive genomes with multiple polymorphic chromosome rearrangements, polyploidy, selfing, and the absence of a genome sequence which allows markers to be easily ordered. There is therefore a requirement for software which works well for plants and data sets in which some markers may be mapped and some not.

### Reference population design

The biggest problem in implementing GS is the design of the RP. Simulations (Clark et al. 2011; Hickey et al. 2014) and empirical studies (VanRaden et al. 2009; Habier et al. 2010; Clark et al. 2012) have demonstrated that the RP should be large; several thousand individuals ideally for an inbreeding cereal (Hickey et al. 2014), though much smaller populations can be used when candidates and RP are very closely related. Initial implementation of GS in a breeding program may, therefore, be best within a very closely related genetic pool, where linkage disequilibrium is extensive, allowing accurate predictions from small numbers of markers and individuals. However, to exploit the full potential of GS, larger populations and markers densities are required.

## Examples of Trait Prediction in Wheat

We give some examples of the accuracy of genomic prediction from work in United Kingdom and European wheat in Table 1. Each example illustrates the success of the process, but also the diverse ways in which reduced relationships between test and RP can greatly reduce the accuracy of prediction.

**Table 1:** Genomic prediction in wheat

Data source	Case 1	Case 2	RP size	No. markers
1. UK NL/RL 1948–2007	0.8	0.2	80	217
2. AxC yield data	0.5	0.2	100	351
3. Triticeae Genome	0.5	0.3	376	1804

Data source: See text for details.

Case 1: Data partitioned at random into test and reference populations.

Case 2: Data partitioned selectively: for details see text.

Example 1 uses historical records of wheat yields from 1948 to 2007, reanalyzed by Mackay et al. 2011). High accuracies are achieved when lines are partitioned into test and reference sets with equal representation of lines from the whole time series (case 1). However, when lines are partitioned into an older set in the RP and modern lines in the test set (case 2), the correlation drops from 0.8 to 0.2, though for lines released within 10 years of the youngest line in the reference set the correlation is 0.4 (Mackay et al. 2011). Case 2 mimics more closely what breeders desire by predicting forward over generations. In this data set, variety yields have increased over time and marker allele frequencies have changed over time. In essence, markers give a good prediction of age and age gives a good prediction of yield, but this fails if the full age range is not represented in the RP.

In the second example, yield and marker data from the publically available doubled haploid mapping population Avalon x Cadenza (http://www.wgin.org.uk/) were partitioned at random into test and RPs (case 1). The cross‐validation correlation of 0.5 is acceptable. However, if the lower yielding lines are used as the reference set to predict the yield of the higher yielding set the correlation drops (case 2). Once more, case 2 mimics more accurately breeders' requirements: to predict varieties with better performance than the best currently available.

The final example uses the TriticeaeGenome association mapping panel (Bentley et al. 2014, http://www.triticeaegenome.eu/ ie. no hypen) of 384 French, German, and UK winter wheat lines, genotyped with DArT markers. If test and reference sets are created without reference to country of origin, then the cross validation correlation is acceptable (case 1). However, if lines from one country of origin are used to predict performance of lines from another (case 2), the correlation drops. Predicting across a greater genetic distance, measured here by country of origin, is more in line with the needs of the plant breeder.

These three examples show that differences between test and RPs in age of varieties, in yield, or in country of origin can all have a major effect on the accuracy of predictions. In all examples, the underlying cause of the reduction in prediction accuracy is the increased genetic distance between lines in the test and RPs. All three also give reasonable examples of what breeders would like to do: predict forward in time, predict transgressive segregation and predict into differing pools of germplasm. In every case, the composition of the RP is key.

## High Throughput Field Phenotyping

Ideally, RPs should be large, closely related to the candidates for selection and accurately phenotyped. These requirements are antagonistic. Accurate phenotyping generally requires multiple replicates of large plots. However, large RPs limit replicate number, and the requirement to multiply seed over several years for trials may cause selection candidates to be several generations removed from the RP. There is a requirement, therefore, to increase the precision with which yields are estimated from low replicate, large entry number yield trials.

### Trial design and analysis

To date, the principle mechanism for increasing precision has been through improvements in trial design. Sophisticated designs are now readily available (e.g., http:/ www.expdesigns.co.uk/co.uk/, http://www.austatgen.org/software/). These have largely removed the constraints on permissible combinations of block size, variety number and replicate number present in old published catalogs of designs in classic texts such as Cochran and Cox (1957). Augmented (Federer 1956), p‐rep (Cullis et al. 2006), and augmented p‐rep trial designs (Williams et al. 2011) warrant mention as they are targeted at very low replicate experiments such as early generation breeders' trials. They are therefore also highly suitable for testing large RPs for GS. Trials which incorporate known genetic relationships among varieties in their arrangement in the field will also help improve precision (Moehring et al. 2014). However, there is a limit to the increased precision that trial design alone can deliver.

An old but little used method of increasing precision is through the analysis of covariance (Fisher 1934; Wishart 1950). Here, adjustment for field environmental effects (on yield, say) is made through the correlation between yield and some other measurement made on the plots or plants. The additional measurement should have no genetic link to yield, but can still be a phenotype. For example, adjustment could be made for plant vigour if it acted as a surrogate for plot fertility. This approach has parallels with genomic selection, where any relationship between trait and markers is purely genetic in origin so selection on marker genotype is indirect selection on G: the trait genotype. In the analysis of covariance, any relationship between the covariate and trait is treated as purely environmental in origin so selection on the covariate is indirect selection on E: the environmental effect. Selection to reduce E then acts to increase the accuracy of G. However, in many cases, covariates also have a genetic correlation with yield. In the case of plant vigour, the analysis of covariance could result in the selection of less vigorous lines. Partly for this reason, analysis of covariance has been little used in plant breeding, and mainly in disaster recovery: when the covariate could be poor field emergence for example. Moreover, routine scoring of additional traits for potential use as covariates is expensive and time consuming, particularly in breeders' large early generation trials. However, the recent introduction of automated methods and platforms to score very large numbers of traits on field plots (Montes et al. 2007; Araus and Cairns 2014) makes their collection cost effective and we believe the routine application of these methods to variety trials should be re‐examined.

Using currently available technology for field phenotyping and for precision agriculture, large numbers of covariates can be recorded quickly at plot level. Many, if not most of these, will correlate with both environmental and genetic determinants of yield so cannot be incorporated into trait prediction in the same way as markers in GS or as environmental covariates in the analysis of covariance. However, using methods originally employed in creating “selection indices” (Smith 1936; Hazel and Lush 1942; Henderson 1963) to select optimally across multiple correlated traits of varying economic importance, it is possible to include these in estimation of yield by taking into account their environmental and genetic covariances with each other and with yield.

### Selection index

A selection index is a linear combination of variables that is used to compute, for each individual or line, a criterion for selection (Henderson 1963). To compute such an index, estimates of genetic and environmental variances and covariances are required among all traits. These estimates are possible from all well designed field trials provided there is some, possibly incomplete, replication, and/or by taking into account genetic relationships among lines estimated by pedigree or by genetic markers (Astle and Balding 2009; Lee et al. 2010). The relative importance of each trait is also required. This is referred to as the trait's “economic value” and is measured as the cash value of a unit increase in the trait. Estimating economic values across multiple traits can be complex, but with only a single‐target trait for selection (say yield) it is simple: the economic value of the trait is 1 (or −1 if we are selecting for decreasing values) and the economic value of all other traits is 0. For completeness, we give the equation to compute the coefficients of the selection index below:

(1) $$b={\mathrm{GP}}^{-1}e$$

where **b** = the vector of regression coefficients to be estimated (including for yield itself), **e** = the vector of economic values (1 for yield and 0 for all other traits and covariates), **G** = the genetic (co)variance matrix, **P** = the phenotypic (co)variance matrix. Excellent accounts of the theory of selection indices are given in Falconer and Mackay (1996) and Bulmer (1980). Once the coefficients are estimated, then for each line each trait in turn is multiplied by its corresponding value in **b** and these are summed to give the value of the selection index for that line. Selecting on this index will give a greater response than selecting on the trait alone and this increase can be predicted (Falconer and Mackay 1996). In Table 2, we give an idealized example with one target trait, Y, and one additional trait, X. The heritability of each trait is fixed at 0.5 and, for ease of interpretation, each trait has the same variance. The correlation between the traits may be all genetic, all environmental or made up of both environmental and genetic effects.

**Table 2:** Selection indices to increase trait Y incorporating data from a second trait X

Phenotypic correlation	Cause of correlation	Selection index	Relative response^a^
0.5	All genetic	Select on Y + X	1.155
0.5	All environmental	Select on Y − X/2	1.155
0.5	Genetic = environmental	Select on Y	1.000
0.0	No cause; genetic = environmental	Select on Y	1.000
0.0	Genetic = ‐ environmental = 0.5	Select on Y + X/2	1.118

Heritability of X = heritability of Y = 0.5 in all cases. Genetic variance = 1.

a Expected response to selection on the index relative to direct selection on Y.

The relative strengths and signs of the genetic and environmental correlation determine the nature of the index: X is selected for if the correlation is all genetic but against if it is all environmental. If the two correlations are equal, then there is no gain to be made by including the second trait. The expected gain in response from selection on the index is substantial for the other examples. Note that the phenotypic correlation may be 0 because there is neither genetic nor environmental correlation, or because the genetic and environmental correlations are of opposite sign and cancel. In the first case, the best selection strategy is to select on Y alone, but in the second some weight is given to X. This emphasizes the importance of decomposing phenotypic correlations into genetic and environmental components. The Excel spreadsheet used to compute the coefficients given in Table S1 is given in the supporting information and can be used to compare coefficients for other values of the parameters, given in equation 1.

Differences in sign of genetic and environmental correlations (*r*_g_ and *r*_e,_ respectively) do occur. Table 3 gives an example from the TriticeaeGenome dataset (Bentley et al. 2014) of correlations between height and flowering time in five trials. The correlations vary from site to site, but in general the environmental correlation is positive and the genetic correlation is negative. A positive environmental correlation between height and yield is most simply interpreted as an indicator of soil fertility: fertile plots yield more and the plants grow taller. The negative genetic correlation is most likely due to the presence in this association mapping panel of older lines, which tend to be taller, carrying no semi‐dwarfing alleles, and modern lines which are shorter and yield more. Table 3 also lists the heritabilities of the two traits and the relative merit of selecting on an index to improve yield by taking into account the additional information provided by height. In this example, the improvement is slight except for data from France in 2010 and the United Kingdom in 2011. At the 2011 United Kingdom trial, seedling establishment, flowering time and tiller number were also scored. If these traits are incorporated into the index, selection is predicted to be 11% more efficient than selecting on yield alone. In breeders' trials, the heritabilities for yield are commonly lower than the high values given here and the scope for improved precision from use of an index is therefore greater.

**Table 3:** TriticeaeGenome trials 2010–2011: 387 wheat varieties of French, German and UK origin

Correlations between yield and height					
Trial location	*r*_e_	*r*_g_	*h*_2_ *y*	*h*_2_ *x*	*RM*
Fr 2010	0.03	−0.45	0.43	0.91	1.074
Ge 2010	−0.07	−0.18	0.59	0.91	1.003
Ge 2011	0.36	−0.13	0.61	0.91	1.009
UK 2010	0.24	−0.03	0.30	0.82	1.008
UK 2011	0.48	−0.24	0.44	0.69	1.066
Average	0.2	−0.2	0.5	0.9	1.03

*r*_e_: Environmental correlation coefficient.

*r*_g_: Genetic correlation coefficient.

*h*_2_ *y*: Heritability of yield.

*h*_2_ *x*: Heritability of height.

*RM*: Relative merit of index selection.

### Combining information from multiple sources

Additional trait and phenotype information collected on the RP can be used to create a selection index for any key traits(s). Genetic markers will be used to generate a prediction equation for that index. Subsequently, candidates for selection will be genotyped and their selection index, say for yield, predicted from that score. The additional gain to come from the use of an index needs to be tested empirically and must compensate for the cost of collection. For our own trials, our current best estimate is that the additional phenotyping adds 28% to the cost of a trial. This estimate is based on actual costs within the GplusE project (described below) in which a 2 × 6 m plot for assessing yield is £18.67, including drilling, harvesting and land rental, and the cost of gathering multiple spectrophotometric readings from six unmanned aerial flights together with ground‐based readings for calibration is £5.25 per plot. Statistical methods are needed to incorporate very many plot covariates into a selection index: the classical selection index equation will only work if there are more varieties in trial than there are additional measurements and the measured traits are not very highly correlated (i.e., there is no colinearity among the measurements). This is analogous to the position with genomic prediction prior to the introduction of statistical methods to circumvent the problem (Whittaker et al. 2000; Meuwissen et al. 2001). Some methods are already available to account for these problems (Hayes and Hill 1980, 1981; Tai 1989) but borrowing and adapting methods from genomic selection may be more effective.

## The GplusE Project

**Figure 1:**
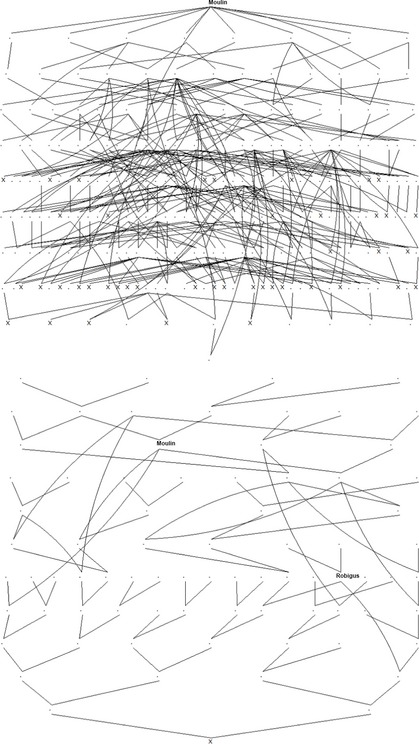
Partial pedigree of 44 crosses selected for GplusE project. Crosses are represented by “X”. Upper panel: descendents of the variety Moulin (listed 1984), the most recent common ancestor of 43 out of 44 of the selected crosses. Lower panel: ancestors of an arbitrarily selected cross. The positions of Moulin and Robigus, a recent ancestor of many of the lines on the current UK recommended list, are identified. Plots were created using Pedigree Viewer (Kinghorn 1994).

In our study, with the collaboration of four breeders – Elsoms, KWS, Limagrain and RAGT – we are creating a large population of at least 3000 wheat lines from up to 44 elite crosses. Representative views of the linked pedigree are given in Figure 1, and illustrate the consanguineous, unstructured and intermeshed nature of the UK winter wheat pedigree. Allele frequencies at genetic markers have changed over time in the United Kingdom (White et al. 2008), but genetic variation has not declined; it increases in periods when more independent breeding programs contribute varieties. The 44 crosses have been selected to cover the diversity of parents that breeders are currently using. The lines we generate will be phenotyped over 2 years and genotyped with the 35 k UK Affymetrix SNP chip (http://www.affymetrix.com/). The large size of the population, and the known pedigree structure, will allow its partition into RPs and test populations with varying degrees of relationship between the two to assess how accuracy of prediction varies with genetic distance and marker density. This approach has been simulated for wheat by Hickey et al. (2014) and the results of those simulations have been used in the experimental design of GplusE. For example, at one extreme, the reference and training populations can be constructed so that for every cross, there are representatives in both. Alternatively, the two populations could be selected to minimize the pedigree relationship between them. This might involve ensuring that they contain no common grandparents or more distant relationships. In between these extremes are partitions where, say, if two crosses have a single parent in common, then lines from one are allocated to the test population only and lines from the other to the reference population only, while individuals from the same cross are never represented in both. There are many other possible partitions, and selection can also take place using marker‐based estimates of kinship rather than those from the pedigree. Superimposed on these alternatives, the size of the reference population can be varied. In comparing results from these alternatives, we hope to understand how to construct the most effective RP for the germplasm which UK breeders are currently using.

Marker imputation algorithms and software will be developed specifically for use in crops, building on early work which is already in routine use in commercial animal breeding programs (Hickey et al. 2012). The contemporary and highly commercially relevant composition of this population will assist in establishing GS for UK wheat as a cost effective strategy for our commercial partners. This collaboration is unique in the level of cooperation required among competitor companies and the degree of goodwill shown in releasing pedigree information of elite crosses to academic collaborators: a measure of the importance with which GS is viewed in the breeding community.

**Figure 2:**
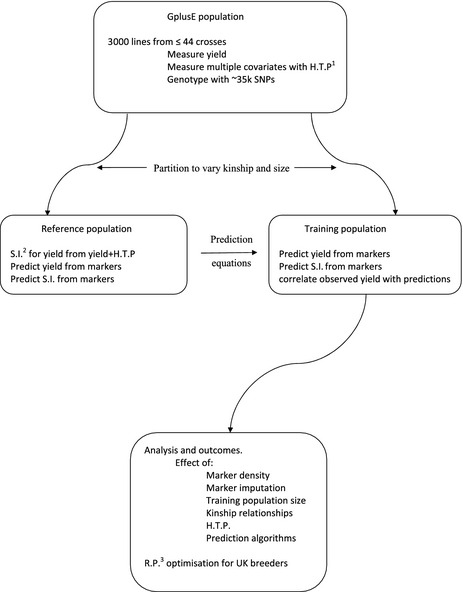
Outline workflow for GplusE. Marker and trait data on the full population are partitioned into a reference population of variable size and a test population with a minimum size of ~300 lines. The reference population can be varied in size and selected to alter kinship relationships among its members and with members of the training population. One or more selection indices are constructed for yield by incorporation of data from high throughput phenotyping. These are then regressed onto a genome wide marker set to create prediction equations which are tested in the training population by comparing observed yield with predicted yield and with the predicted selection indices. All combinations of parameters will be tested in replicated cross‐validations. ^1^H.T.P.: high thoughput phenotyping. ^2^S.I.: selection index. ^3^R.P.:reference population.

In addition to the study of RP composition for predicting yield, additional covariate and trait information will be collected. Data on soil composition at the plot level will be collected by ground‐based electromagnetic induction (EMI) by SOYL Precision Farming. (http://www.soyl.com/). EMI is used in precision agriculture to measure various soil properties, including topsoil depth, soil texture and moisture content (Doolittle and Brevik 2014). Soil penetrometer measurements will be used as a proxy for root activity in surface soil layers (Whalley et al. 2008). Airborne multispectral reflectance signatures (visible and infra red) will be captured at the plot level by Ursula Agriculture (http://www.ursula-agriculture.com/). Ground‐based spectral reflectance measurements will be used in combination and to inform targeted airborne data captured through the use of the UAS (Unmanned Aerial System). We plan to score the trial from the ground three times and from the air six times between sowing and harvest, though this partition may vary subject to initial results. UA have proprietary algorithms to predict biomass, lodging potential, establishment, vigour, crop cover, pests, diseases, weeds, and stress. These will be included, but it is possible that the raw reflectance scores function better as covariates in creating an index for selection: there is no requirement in the construction of a selection index for the use of these covariates to have a biological interpretation any more than there is a requirement in genomic prediction to select subsets of markers which tag statistically significant QTL. This will be tested: First selection indices will be constructed in the reference population from yield and either the raw reflectance scores or from yield and the traits derived from those scores. Then, within the RP, yield and the selection indices with be regressed on the markers to derive prediction equations for each. The data in the test population will then be used to compare observed yield with yield predicted from the three different prediction equations (recalling that the selection indices are merely predictors of yield). A simplified schematic of the analysis pipeline is given in Figure 2.

Our population will be grown in augmented p‐rep trials at two sites in each year (though additional testing may occur outside the scope of the project). The trials protocol will otherwise follow standard practice in the United Kingdom for fungicide‐treated trials, as described for running the UK recommended list system (http://www.hgca.com/). Using standard breeders' trial plot sizes of 2 × 6 m, this means that each trial is ~4 ha. No fixed field‐phenotyping platform could cover this area: those currently available commercially are prohibitively expensive and might contain 80 plots, at best. For GplusE, the requirement is to gather additional trait and covariate information cheaply on a large scale. Our choice of field phenotyping platforms was dictated by cost, throughput and availability. The potential of automated phenotyping to add value to large field experiments is great and several high throughput ground‐based and aerial systems now exist or are in development (Araus and Cairns 2014). Although GplusE is focussed on developing and enhancing GS of UK wheat, the data the project generates will have other uses. The 44 crosses in the RP are linked by pedigree and can be used in linkage analysis and join linkage disequilibrium linkage analysis (LDLA) (Meuwissen and Goddard 2001) to detect QTL of larger size. The field phenotyping data can be used directly to develop and test physiological hypotheses. Moreover, if the methods and algorithms we describe work, these can be applied to any field experiment to improve the precision of the assessment of the primary outcome (commonly yield). We look forward to a time when additional covariate and trait information is collected routinely in any field experiment; it can be incorporated as easily into fertilizer or pesticide trials as into variety trials.

Although we have focused on yield, bread making quality or any other quantitative trait is amenable to the GplusE approach. Limited progress in improving the quality of UK wheat can be regarded as a “market failure” (Galushko and Gray 2014): the return to the commercial breeder from improving this trait does not warrant the investment required. It is possible that the cost of breeding could be reduced substantially if marker‐based prediction of quality was substituted for direct phenotypic assessment. The cost of phenotyping the reference population cannot be avoided, but its accuracy may be increased by applying the selection index methods described above to combine the currently used array of predictive tests of grain quality (e.g., Cavanagh et al. 2010) into an index. Genomic selection could then be based on this index. A similar approach has been described by Heffner et al. (2011). Nevertheless, genomic selection for improved quality may require restriction to selection among individuals very closely related to the RP – in the extreme the candidates and the RP could all come from a single cross. In these circumstances, the size of the training population can be reduced substantially.

## Conclusion

Crop genetics is becoming data rich as the technologies underpinning the various new ‘omics disciplines (genomics, metabolomics, phenomics etc.) are applied. Many new biological insights will emerge. Crop improvement through plant breeding, however, will remain the major route by which targets of sustainable intensification will be achieved. Currently, GS is the most promising way in which improvements in polygenic traits such as yield will be made. This process of translating high throughput genetic marker systems into breeding practice is well developed in animal breeding and is beginning to be applied in crops. GplusE will assist in this process for UK wheat by increasing understanding of the dynamics of RP design and by testing if data from high throughput field phenotyping can be integrated directly into the process. Success would pump‐prime the routine application of GS for UK wheat breeding, which could also improve the precision of all future field experimentation.

## Conflict of Interest

None declared.

## Supporting information


**Table S1**. Selection indices to increase trait Y incorporating data from a second trait X.
