# Supplementary material for: GplusE: beyond genomic selection
Source: Food Energy Secur. 2015 Mar 25;4(1):25–35. doi: 10.1002/fes3.52 (PMC4998132; doi:10.1002/fes3.52)
Supplement: Supplementary file 1 — Table S1. Selection indices to increase trait Y incorporating data from a second trait X. [file FES3-4-25-s001.docx]

Table S1 Selection indices to increase trait Y incorporating data from a second trait X.

**Phenotypic**

**correlation cause of correlation selection Index relative response^*^**

0.5 all genetic select on Y + X 1.155

0.5 all environmental select on Y - X/2 1.155

0.5 genetic = environmental select on Y 1.000

0.0 no cause; genetic = environmental select on Y 1.000

0.0 genetic = - environmental = 0.5 select on Y + X/2 1.118

Heritability of X = heritability of Y = 0.5 in all cases. Genetic variance = 1.

^*^ expected response to selection on the index relative to direct selection on Y.
